# Regulatory technology arrangements for hospital bed access during the COVID-19 pandemic: scope review

**DOI:** 10.1590/0102-311XEN044824

**Published:** 2025-06-27

**Authors:** Mariana Prado Freire, Arthur Chioro, Fernando Tureck, Luís Fernando Nogueira Tofani, Letícia Gabriela da Silva, André Luiz Bigal, Amanda da Cruz Santos Vieira, Marília Cristina Prado Louvison

**Affiliations:** 1 Faculdade de Saúde Pública, Universidade de São Paulo, São Paulo, Brasil.; 2 Universidade Federal de São Paulo, São Paulo, Brasil.; 3 Universidade Metropolitana de Santos, Santos, Brasil.; 4 Universidade do Contestado, Mafra, Brasil.; 5 Faculdade Santa Marcelina, São Paulo, Brasil.

**Keywords:** Health Care Coordination and Monitoring, Access to Health Services, COVID-19, Hospital Bed Capacity, Regulación y Fiscalización en Salud, Acceso a los Servicios de Salud, COVID-19, Capacidad de Camas en Hospitales

## Abstract

The COVID-19 pandemic posed numerous challenges to health care systems, including hospital bed access. Analyzing the response provided and identifying regulatory technology arrangements that contributed to provide improved health care access can support the preparation of systems for future emergencies and also provide alternatives to current difficulties. The aim of this study is to mapping, summarize and categorize the regulatory technology arrangements used in hospital bed access during the COVID-19 pandemic using the scoping review method. The review used the recommendations of the Joanna Briggs Institute and the PRISMA-ScR report, and the PCC acronym (population [P]: of COVID-19 or non-COVID-19 patients; the concept [C]: of bed access regulation; and the context [C]: of the COVID-19 pandemic). The search was carried out in the PubMed, Scopus, Embase, Web of Science and LILACS databases between July and September 2022 and 45 articles were selected for review. We established three categories of analysis: Reorganization of services; Use of virtual tools and artificial intelligence; and Creation of alternative spaces, that assisted in the identification and analysis of the regulatory technology arrangements used. We note arrangements such as telehealth and telemedicine, the reconfiguration of existing spaces for expansion of health care capacity and modifications in the work process, including the adoption of specific protocols and the establishment of differentiated flows. The review showed the importance of regulatory technology arrangements in bed access and challenges such as regulation and financing, factors that may be essential for the use of regulatory technology arrangements.

## Resumen

La pandemia de la COVID-19 ha generado numerosos desafíos a los sistemas de salud, como el acceso a camas hospitalarias. La evaluación de la respuesta brindada y la identificación de las disposiciones regulatorias tecnológicas que contribuyeron al acceso a la atención pueden cooperar en la preparación de sistemas para futuras emergencias y también ofrecer alternativas a las dificultades actuales. El objetivo de este estudio es mapear, sintetizar y categorizar las disposiciones regulatorias tecnológicas utilizadas para acceder a las camas hospitalarias en el contexto del afrontamiento de la pandemia de la COVID-19 mediante el método de revisión de alcance. La revisión contó con las recomendaciones del Instituto Joanna Briggs y con el informe PRISMA-ScR, y las siglas PCC (populación [P]: pacientes con COVID-19 o sin esta enfermedad, el concepto [C]: regulación de acceso a camas y el contexto [C]: pandemia de la COVID-19). La búsqueda se realizó en las bases de datos PubMed, Scopus, Embase, Web of Science y LILACS entre julio y septiembre de 2022; y se seleccionaron 45 artículos para su revisión. Se determinaron tres categorías de análisis: Reorganización de servicios; Uso de herramientas virtuales e inteligencia artificial; y Creación de espacios alternativos, para ayudar en la identificación y análisis de las disposiciones regulatorias tecnológicas utilizadas. Se destacan las disposiciones como la telesalud y la telemedicina, la reconfiguración de los espacios existentes para la ampliación de la capacidad asistencial y modificaciones en el proceso de trabajo, como la adopción de protocolos específicos y el establecimiento de flujos diferenciados. La revisión indicó la importancia de disposiciones regulatorias tecnológicas en el acceso a las camas y los desafíos como la regulación y el financiamiento, factores que pueden ser críticos para el uso de disposiciones regulatorias tecnológicas.

## Introduction

Due to the global health crisis caused by SARS-CoV-2, a type of coronavirus, on January 30, 2020 the World Health Organization (WHO) declared a Public Health Emergency of International Concern (PHEIC). The goal was to coordinate the response to the new health emergency by releasing a set of recommendations including support for vulnerable health systems and prompt development of vaccines and therapies ^1^.

At the time the WHO declared the PHEIC and recognized the pandemic, on March 11, 2020, new COVID-19 cases and deaths increased, which led to diverse and often disordered health care responses around the world. Evidence indicates that the tracing of needs and the organization of the response to the pandemic, especially during the initial phase, were based mainly on the knowledge acquired when facing other significant health crises and on the existing local health care framework ^2^ ^,^ ^3^. Thus, the pandemic showed the strengths and weaknesses in health care systems, resulting indistinctly in varied and often questionable responses, even in internationally recognized health care systems ^4^.

In addition to the WHO, international organizations such as the U.S. Centers for Disease Control and Prevention (CDC) and the European Center for Disease Prevention and Control (ECDC) released recommendations that served as a scientific basis and practical guideline for the implementation of actions, including measures for health surveillance and service management. Although the guidelines on the implementation of non-pharmacological measures were prioritized during the pandemic, strategies aimed at patient care were emphasized, such as monitoring cases by phone or apps, expanding hospital capacity and implementing telehealth tools ^5^ ^,^ ^6^ ^,^ ^7^.

The first countries affected by the virus served as important benchmarks for preparing the response of health care systems to the pandemic. Italy, for example, recommended staff training, acquisition of equipment and supplies, and designation of teams and areas dedicated to the care of COVID-19 patients ^8^ ^,^ ^9^ ^,^ ^10^ ^,^ ^11^ ^,^ ^12^ ^,^ ^13^. However, compliance with the recommendations of international authorities to increase the number of beds, suspend elective procedures, and increase the number of workers ^2^ ^,^ ^14^ was insufficient to prevent health care demand from surpassing the installed capacity of the services.

Some countries with health care systems - circumstantially under pressure and alerted to the risk of imminent failure - instituted regulating mechanisms that were fundamental for the organization of the response, in an attempt to correct the gaps between supply and demand. Thus, conceptually, health care regulation is the Public Health field that mediates between health needs and availability, intervening in existing market relations in search of a balance between supply and demand ^15^.

Saltman & Busse ^16^ note that, in Europe, the concept of regulation is indicated as a non-linear process built in order to consider a variety of regulating tools that have been established throughout the construction of the health care systems. In the Brazilian context, specifically within the scope of the Brazilian Unified National Health System (SUS, acronym in Portuguese), regulation is understood as a management function including structural aspects of system regulation, the regulation of services, and the regulation of access. The latter is defined as “*the provision of the most appropriate health care alternative according to the needs of citizens, in an equitable, organized, timely and qualified manner, effected through regulatory complexes*” ^17^.

In practice, the bureaucratic characteristics of health care regulation in Brazil often diverge from the daily needs of health care services and citizens, according to the observations of those involved. The political and theoretical constructions related to macro-regulation are not sufficient to guarantee users’ access to health care services, and the regulation of access poses important challenges as to effectively making supply closer to demand ^18^.

Accordingly, the broader concepts of access help in the analysis of regulating processes, extrapolating the geographical or availability dimensions, as found in a review carried out by Travassos & Martins ^19^. In summary, according to the authors, the idea of accessibility of Donabedian, in its socio-organizational dimension, considers the factors that favor or hinder access, as well as the adequacy of human and technological resources to meet the demand ^19^. They corroborate - albeit with conceptual divergences - the contributions of Andersen ^20^, providing a view of access that goes beyond entry into the health care service, including the path and subsequent care required.

Penchansky & Thomaz (1981, *apud* Travassos & Martins ^19^) introduced another refinement of the concept of access called accommodation, which concerns the degree of fit between supply and demand, considering how services are organized to receive patients and how patients receive the care provided. The conceptual polysemy of access favors the analysis of the production of care adopted during the pandemic, considering the variety of care flows produced inside and outside hospitals during the health crisis, including the challenges in relation to successive care, the decision-making processes as to needs and supplies, and the organization of actions.

According to Cecílio et al. ^21^, access regulation creates a situation of conflict, in which actors involved in the process play important roles, going beyond mere “*bureaucratic-administrative ordering*”, involving operating in different “*regulation regimes*”, encompassing the different dimensions of users, workers, managers, providers and others. Consistently, several authors have contributed to reformulate the conception of access regulation as a constantly evolving process, informed by the micropolitics of health care services ^22^ ^,^ ^23^.

In a broader context, when dissociated from actions implemented by bureaucratic and structured processes, access regulation initiatives address the production of care seeking to overcome medical-hegemonic models and open space for different knowledges in the field of public health care. These initiatives, situated in the field of micropolitics of health care work, value the organization of “*living*” work in action and the strength of interpersonal relationships in health care practice ^24^ and have been called regulatory technology arrangements ^22^ ^,^ ^23^. In other words, regulatory technology arrangements are essential tools that contribute to the organization of health care, providing a broader view of access regulation beyond the traditional regulatory complex, contemplating innovative strategies that meet the health needs of the population ^22^.

During the pandemic, with increased demands and extremely insufficient supplies, the central roles of regulation may have shifted and triggered diverse responses. The identification of successful regulatory technology arrangements in the period that contributed to improve access to beds and considered the extraordinary demand and the very high number of deaths ^25^ can be valuable not only for the management of future health crises, but also to provide new models for addressing daily difficulties in the field of regulation. In addition, the resilience of health care systems was essential for fast adaptation to adverse circumstances, maintaining the capacity to respond to unforeseen challenges ^26^. Thus, the objective of this study is to mapping, summarize and categorize the regulatory technology arrangements used in hospital bed access during the COVID-19 pandemic, including developments in outpatient care and non-covid care, when related.

## Methodology

This review is part of the research funded by the São Paulo State Research Foundation (FAPESP, acronym in Portuguese) with resources from the SUS Research Program (PPSUSCovid, acronym in Portuguese), entitled *Confronting the COVID-19 Pandemic: Productions, Inventions and Challenges in Networked Care Management* [Enfrentamento da Pandemia de COVID-19: Produções, Invenções e Desafios na Gestão do Cuidado em Rede]. The need to conduct a review on the subject was found during the research, in order to mapping the existing academic production on the subject in a comprehensive manner, enabling the observation of reality as a basis for understanding the research findings.

Therefore, the scope review method was chosen because it can show concepts and definitions and identify factors related to the concept ^27^. The methodology was built according to the recommendations of the Joanna Briggs Institute for scope reviews and the report *Preferred Reporting Items for Systematic Reviews and Meta-Analyses: Extension for Scoping Reviews* (PRISMA-ScR), including updates ^28^ ^,^ ^29^.

In line with the main research object, i.e., the identification of arrangements and innovations in addressing the pandemic, the research question was built accordingly, being: “What regulatory technology arrangements for bed access regulation were produced during the pandemic?”. After defining the research question, the search strategy was built based on the PCC acronym (population, concept, context), with population (P) including with COVID-19 and non-COVID-19 patients, concept (C) referring to bed access regulation, and context (C) covering the COVID-19 pandemic.

The search strategy was developed based on the MeSH (https://www.ncbi.nlm.nih.gov/mesh/) and DeCS (https://decs.bvsalud.org) platform descriptors to cover a comprehensive spectrum of studies. The search terms used were “*covid-19*” *AND* “*acesso à saúde*” *AND* “*regulação em saúde*”, in Portuguese, and “*covid19*” *AND* “*delivery of healthcare*” *AND* “*regulation in health*”, in English. Considering the particularity of the application of the term “*regulação em saúde*” in Portuguese, but specifically in the Brazilian literature, calibration steps were necessary, using terms indexed with different combinations in the main databases to verify their ability to express the reality under research.

After defining the terms, the searches were carried out in the PubMed, Scopus, Embase, Web of Science and LILACS databases, which were chosen to ensure the coverage of the journals indexed under the subject; the searches were performed from July to September 2022, comprising the years from 2020 to 2022, in the context of the COVID-19 pandemic.

The Rayyan platform (https://www.rayyan.ai/) was used for screening ^30^, analyzing titles and abstracts, applying the eligibility criteria established based on the PCC acronym. We excluded articles not directly related to addressing the pandemic, not addressing access to hospital or emergency room beds, and opinion or prescription articles. The review considered only studies that described the regulatory technology arrangements implemented during the pandemic for the care of COVID-19 and non-COVID-19 patients, reviews on the same subject, and did not apply geographical limitations. In addition, articles available in languages other than Portuguese, Spanish and English were excluded.

The articles considered eligible were reinserted in the Rayyan platform and evaluated in full by the reviewers, using the established exclusion criteria, and a data extraction matrix was built containing the relevant information to be collected in the articles, including identification data (location, year, language, authors, title, journal) and analytical data (type of study, objectives, identified arrangements, target population and results achieved with the implementation of the arrangement, when described).

The results were analyzed and systematized using Content Analysis techniques ^31^, based on pre-established categories of analysis. After exhaustive processing and coding of the material, the existing categories were refined and subcategories of analysis were created, which enabled the grouping and identification of contents related to the most relevant themes found on the subject in the literature.

The team of researchers held 13 online meetings, using the Google Meet platform (https://meet.google.com), where all stages of the review were discussed: conception, identification of search terms, alignment of inclusion and exclusion criteria, data extraction and data analysis. The virtual meetings were also used for collective analysis of certain articles to obtain consensus.

## Results

**Figure 1:**
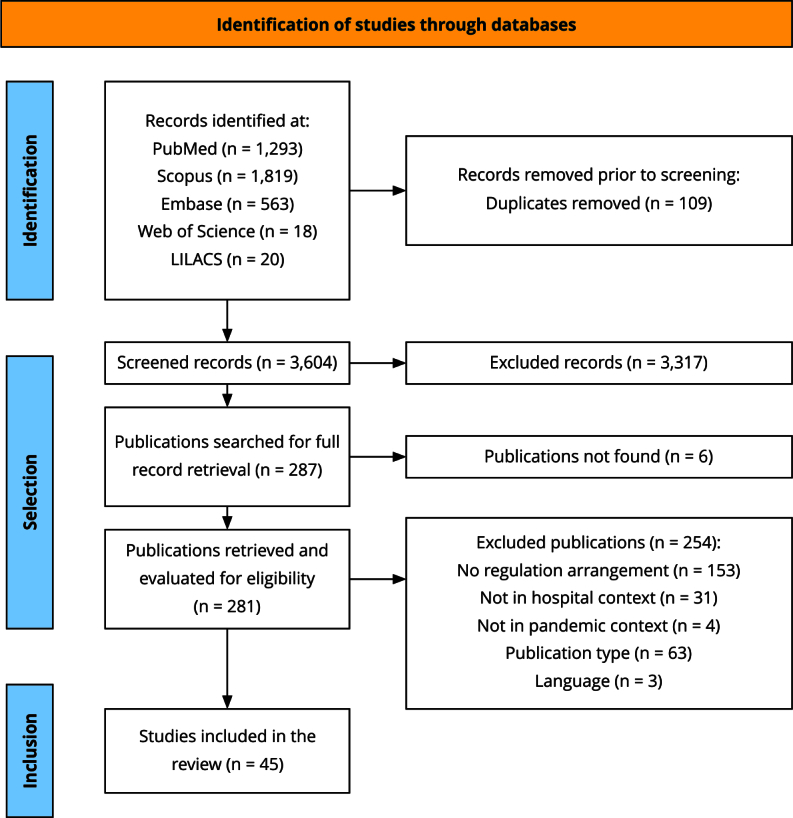
Study identification, selection and inclusion flowchart.

The search in the databases resulted in 3,713 records, of which 109 were duplicates and were eliminated, leaving 3,604 records for the first stage. We considered 281 records eligible for full reading and application of the established exclusion criteria, using the Rayyan platform. Most of the excluded records did not meet the criteria related to the concept of hospital bed access regulation (60.2%), followed by studies outside the scope of publication (24.8%), outside the research context (12.2%), outside the pandemic context (1.5%), and in a language not included (1.2%). After rigorous application of the exclusion criteria, 45 records were included in the review, as shown in Figure 1.

The articles included, detailed in Box 1, are distributed geographically, with a predominance of studies carried out in the United States, representing 37.7% (17 articles), followed by European literary production, representing 35.5% of the total (16 articles), noting Italy (7 articles). Nine articles from the Asian continent were included, representing 20% of the total, and South Korea contributed with one third of the total articles, in addition there was 1 Brazilian article and 1 Australian article, as well as a literature review that has global coverage; however, 95% of the articles found are in English.

**Box 1:** Studies included in the review.

STUDY	TITLE	LANGUAGE	COUNTRY	PUBLICATION YEAR	STUDY TYPE
Fuchs et al. ^41^	*Assessment of a Hotel-Based COVID-19 Isolation and Quarantine Strategy for Persons Experiencing Homelessness*	English	United States	2021	Retrospective cohort study
Bruni et al. ^43^	*Telemedicine-Enabled Accelerated Discharge of Patients Hospitalized with COVID-19 to Isolation in Repurposed Hotel Rooms*	English	Italy	2020	Experience report
Kang et al. ^39^	*Operating Protocols of a Community Treatment Center for Isolation of Patients with Coronavirus Disease, South Korea*	English	South Korea	2020	Descriptive study
Paganini et al. ^40^	*Implementation of a nurse-led alternate care site for the management of the surge of patients with COVID-19 in an Italian emergency department*	English	Italy	2022	Retrospective study
Bar-On et al. ^42^	*Establishing a COVID-19 Treatment Centre in Israel at the Initial Stage of the Outbreak: Challenges, Responses and Lessons Learned*	English	Israel	2021	Retrospective study
Lee et al. ^59^	*COVID-19 Level-Loading: Transferring Emergency Department Patients to a Partner Academic Medical Center Within a Healthcare System*	English	United States	2021	Experience report
Lowe et al. ^33^	*Avoiding Crisis Conditions in the Healthcare Infrastructure: 2 Case Studies in Statewide Collaboration*	English	United States	2022	Multiple case study
Ranzani Rigotti et al. ^34^	*Resilience of Healthcare Systems in the Face of COVID-19: An Experience Report*	Portuguese/English	Brazil	2022	Experience report
Longo et al. ^54^	*Managing Head and Neck Cancer Patients During the COVID-19 Pandemic: The Experience of a Tertiary Referral Center in Southern Italy*	English	Italy	2021	Experience report
Shrestha et al. ^35^	*Anesthesiology and Critical Care Response to COVID-19 in Resource-Limited Settings: Experiences from Nepal*	English	Nepal	2021	Experience report
Al Garhy et al. ^70^	*Mental Health Services During the COVID-19 Pandemic in Abu Dhabi, UAE*	English	United Arab Emirates	2021	Case study
O’Rielly et al. ^51^	*Surgery and COVID-19: A Rapid Scoping Review of the Impact of the First Wave of COVID-19 on Surgical Services*	English	Global	2021	Scoping review
Di Bidino & Cicchetti ^25^	*Impact of SARS-CoV-2 on Provided Healthcare. Evidence from the Emergency Phase in Italy*	English	Italy	2020	Realistic literature review
Burden et al. ^55^	*The Provision of a Time-Critical Elective Surgical Service During the COVID-19 Crisis: A UK Experience*	English	England	2021	Experience report
Bunch et al. ^56^	*Immuno-Thrombotic Complications of COVID-19: Implications for Timing of Surgery and Anticoagulation*	English	United States	2022	Experience report
Mathews et al. ^14^	*Variation in Initial U.S. Hospital Responses to the Coronavirus Disease 2019 Pandemic*	English	United States	2021	Observational study
Lee et al. ^67^	*Clinical Pathway for Emergency Brain Surgery During COVID-19 Pandemic and its Impact on Clinical Outcomes*	English	South Korea	2021	Retrospective study
Lefrant et al. ^2^	*A National Healthcare Response to Intensive Care Bed Requirements During the COVID-19 Outbreak in France*	English	France	2020	Retrospective multicenter observational study
Burgansky et al. ^52^	*COVID-19 in a Community Hospital*	English	United States	2020	Description
Archer et al. ^66^	*Prioritizing Critical-Care Resources in Response to COVID-19: Lessons from the Development of Thailand’s Triage Protocol*	English	Thailand	2020	Protocol
Gagliano et al. ^5^	*COVID-19 Epidemic in the Middle Province of Northern Italy: Impact, Logistics, and Strategy in the First Line Hospital*	English	Italy	2020	Case study
Giorgi et al. ^49^	*The Management of Emergency Spinal Surgery During the COVID-19 Pandemic in Italy*	English	Italy	2020	Experience report
Samofalov et al. ^36^	*Information And Communication Technologies In Public Management of the Healthcare Institutions Network During COVID-19 Pandemics*	English	Poland	2020	Descriptive study
Settelmeier et al. ^71^	*Capacity Changes in German Certified Chest Pain Units During COVID-19 Outbreak Response*	English	Germany	2020	Observational study
Oakley et al. ^37^	*Assembly Line ICU: What the Long Shops Taught Us about Managing Surge Capacity for COVID-19*	English	England	2020	Ecological study
Piazza et al. ^57^	*The Challenge of Maintaining Necessary Vascular and Endovascular Services at a Referral Center in Northern Italy During the COVID-19 Outbreak*	English	Italy	2021	Retrospective ecological study
Fraymovich et al. ^72^	*A Blueprint for Pediatric Emergency Resource Reallocation During the COVID-19 Pandemic: An NYC Hospital Experience*	English	United States	2020	Retrospective ecological study
Hartford et al. ^60^	*Pediatric Emergency Department Responses to COVID-19: Transitioning from Surge Preparation to Regional Support*	English	United States	2020	Descriptive study
Cuschieri et al. ^58^	*The COVID-19 Pandemic: Lessons Learned for Sustained Trauma Preparedness and Responses*	English	United States	2021	Descriptive study
Wood et al. ^38^	*The Value of Triage During Periods of Intense COVID-19 Demand: Simulation Modeling Study*	English	United Kingdom	2021	Description/Case study
Hinson et al. ^73^	*Multisite Implementation of a Workflow-Integrated Machine Learning System to Optimize COVID-19 Hospital Admission Decisions*	English	United States	2022	Experience report
Blazey-Martin et al. ^74^	*Primary Care Population Management for COVID-19 Patients*	English	United States	2020	Implementation evaluation
Kesavadev et al. ^44^	*A New Interventional Home Care Model for COVID Management: Virtual Covid IP*	English	India	2021	Experience report
Bircher et al. ^75^	*Remote Care and Triage of Obstetric Patients with COVID-19 in the Community: Operational Considerations*	English	United Kingdom	2022	Experience report
Panagopoulos et al. ^45^	*Intelligent Pervasive Monitoring Solution of COVID-19 Patients*	English	Greece	2022	Experience report
Greven et al. ^76^	*Telemedicine in Spine Surgery: Outcomes for 138 Patients with Virtual Preoperative Assessment Compared to Historical Controls*	English	United States	2022	Case study-controle
Singh et al. ^69^	*Impact of Telemedicine on Hospitalisation and Mortality Rates in Community-Based Haemodialysis Centres in Singapore During the COVID-19 Pandemic*	English	Singapore	2020	Cohort study
Kim et al. ^46^	*A Brief Telephone Severity Scoring System and Therapeutic Living Centers Solved Acute Hospital-Bed Shortage During the COVID-19 Outbreak in Daegu, Korea*	English	South Korea	2020	Descriptive study
Koziatek et al. ^6^	*Assessing the Impact of a Rapidly Scaled Virtual Urgent Care in New York City During the COVID-19 Pandemic*	English	United States	2020	Retrospective cohort study
Carlberg et al. ^77^	*Preliminary Assessment of a Telehealth Approach to Evaluating, Treating, and Discharging Low-Acuity Patients with Suspected COVID-19*	English	United States	2020	Retrospective study - cohort type
Ferry et al. ^47^	*A Virtual Ward Model of Care for Patients With COVID-19: Retrospective Single-Center Clinical Study*	English	Australia	2021	Retrospective study - cohort type
Borgen et al. ^50^	*From Hospital to Home: An Intensive Transitional Care Management Intervention for Patients with COVID-19*	English	United States	2020	Observational study - cohort type
Kodama et al. ^48^	*Reengineering the Discharge Transition Process of COVID-19 Patients Using Telemedicine, Remote Patient Monitoring, and Around-the-Clock Remote Patient Monitoring from the Emergency Department and Inpatient Units*	English	United States	2020	Retrospective study
Fuentes et al. ^53^	*Impacto de la Pandemia de COVID-19 en la Organización Asistencial del Ictus. Plan Ictus Madrid*	Spanish	Spain	2020	Evalautive study
Lai et al. ^78^	*Digital Triage: Novel Strategies for Population Health Management in Response to the COVID-19 Pandemic*	English	United States	2020	Description

Source: prepared by the authors.

Regarding the year of publication, we observed a higher concentration of articles published in 2020, the first year of the pandemic (22 articles). In 2021, we identified 15 articles, with a proportional reduction in 2022, the last year analyzed (8 articles). Among the studies identified, there was a large portion of experience reports (11 articles), retrospective studies (9 articles), descriptive studies (7 articles) and case studies (4 articles).

During the process of extracting data from the included articles, the identification of regulatory technology arrangements used in hospital bed access was prioritized, since this information is crucial for the answer to the research question, and the objectives of the action and results obtained by applying the described arrangement were also extracted.

Through the foreground analysis, which includes the details of the regulatory technology arrangements used, the target population, objective and results, three categories of analysis were established: (i) Reorganization of services, a topic that covers arrangements directly related to management and workforce; (ii) Use of virtual tools and artificial intelligence, highlighting the use of technology in the implementation of the arrangements; and (iii) Creation of alternative spaces, primarily related to issues involving infrastructure and expansion/modification of physical spaces, as shown in Box 2. Each of the categories consists of a grouping of regulatory technology arrangements for access to beds during the COVID-19 pandemic, which is related to the aspects mentioned above, with the nature of the arrangements being the main element of their categorization. We can also note regulatory technology arrangements that were produced in outpatient care actions, including for non-COVID-19 cases, but these were developments or consequences of actions and arrangements aimed at hospital care and access to beds, thus reducing outpatient care supply in certain situations.

**Box 2:** Analysis categories and subcategories.

ANALYSIS CATEGORY	SUBCATEGORY	DESCRIPTION
Reorganization of services	1.1) Implementation of flows/protocols	Implementation of triage protocol ^5^ ^,^ ^14^ ^,^ ^36^ ^,^ ^40^ ^,^ ^43^ ^,^ ^51^ ^,^ ^56^ ^,^ ^67^
		Implementation of admission protocol/access protocol/COVID-19 protocol ^25^ ^,^ ^40^ ^,^ ^51^ ^,^ ^52^ ^,^ ^54^ ^,^ ^57^ ^,^ ^60^ ^,^ ^66^ ^,^ ^70^
		Implementation of patient transfer protocol ^33^
		Implementation of internal protocols (patient triage) ^35^ ^,^ ^39^ ^,^ ^49^
		Change in internal service flows ^2^ ^,^ ^34^ ^,^ ^36^ ^,^ ^37^ ^,^ ^55^ ^,^ ^58^ ^,^ ^72^
	1.2) Change in supply	Increase in supply (for COVID-19 care) ^2^ ^,^ ^5^ ^,^ ^14^ ^,^ ^33^ ^,^ ^35^ ^,^ ^37^ ^,^ ^39^ ^,^ ^40^
		Decrease in supply - outpatient, elective and non-urgent care - saving human resources, materials and physical space for COVID-19 care ^2^ ^,^ ^5^ ^,^ ^25^ ^,^ ^35^ ^,^ ^51^ ^,^ ^52^ ^,^ ^53^
		Service reorganization to maintain the supply of elective procedures ^42^ ^,^ ^49^ ^,^ ^54^ ^,^ ^55^ ^,^ ^56^ ^,^ ^57^ ^,^ ^58^
	1.3) Work process readjustment	Training, implementation of new clinical practices ^14^ ^,^ ^36^ ^,^ ^37^ ^,^ ^48^ ^,^ ^50^ ^,^ ^51^ ^,^ ^52^ ^,^ ^60^
		Reallocation of professionals ^2^ ^,^ ^53^ ^,^ ^70^
		Readjustment in internal logistics processes ^25^ ^,^ ^51^
		Change in triage model (“reverse triage”) ^43^
		Change in epidemiological data collection to support planning ^5^
	1.4) Structural readjustment	Restructuring of the physical space of the service ^5^ ^,^ ^53^ ^,^ ^58^ ^,^ ^60^ ^,^ ^71^ ^,^ ^72^
		Ward separation ^39^ ^,^ ^42^
		Change in the allocation of physical space to expand supply - transformation of anesthetic recovery rooms, acute ward into spaces for the care of COVID-19 patients ^2^ ^,^ ^14^ ^,^ ^35^ ^,^ ^70^
	1.5) Structuring of transfer/regulation center	Implementation of a transfer center to coordinate patient flow between services ^14^ ^,^ ^33^ ^,^ ^59^
		Appointment of a coordinator responsible for the transfer center - centralizing network coordination and responsibilities ^59^
		Establishment of a system for referral with surrounding hospitals ^39^
	1.6) Request for private beds	Request for private beds, through the establishment of regulations built with political coordination of actors ^33^
Use of virtual tools and artificial intelligence	2.1) Triage/Regulation tool for clinical decision-making support - use of artificial intelligence and information and communication technology	Use of technology for decision-making on patient admission/discharge ^6^ ^,^ ^33^ ^,^ ^73^
		Development and use of an algorithm to predict the severity of cases ^74^ ^,^ ^75^
		Use of artificial intelligence in remote patient triage and follow-up ^48^ ^,^ ^78^
	2.2) Use of telehealth/telemedicine	Use of telehealth/telemedicine for patient admission, follow-up and discharge - “transition of care” to home ^36^ ^,^ ^42^ ^,^ ^48^ ^,^ ^50^ ^,^ ^53^ ^,^ ^54^ ^,^ ^69^ ^,^ ^77^
		Implementation of remote matrix support between hospitals in case management ^36^
		Use of telemedicine in preoperative assessment ^76^
	2.3) Remote patient follow-up	Remote patient follow-up to monitor the evolution of the clinical condition and hospitalization criteria ^36^ ^,^ ^44^ ^,^ ^46^ ^,^ ^47^ ^,^ ^48^ ^,^ ^50^ ^,^ ^75^
		Use of algorithm/artificial intelligence in patient follow-up ^45^ ^,^ ^47^ ^,^ ^74^
		Implementation of self-examination ^40^
	2.4) Creation of virtual ward	Implementation of a virtual ward, including patients in a remote follow-up program after risk assessment and classification ^47^ ^,^ ^50^ ^,^ ^75^
Creation of alternative spaces	3.1) Implementation of community/alternative structure - “Field Hospitals”	Implementation of a community structure to follow-up patients without signs of severity ^39^
		Creation of an alternative space for the care of COVID-19 patients, based on the regional demand ^33^
		Creation of alternative nurse-led space ^40^
		Implementation of an extension structure connected to the hospital for the care of COVID-19 patients, promoting total separation of space and ensuring continuity of non-COVID-19 care ^42^
	3.2) Implementation of hospital beds for COVID-19 patients in hotels with the support of a health care professional	Use of beds for isolation of homeless COVID-19 patients ^41^
		Use of beds to expedite hospital discharge, continuing treatment in beds installed in hotels ^43^

Soruce: prepared by the authors.

The findings in this literature review demonstrate the relevance of the regulatory technology arrangements adopted during the COVID-19 pandemic used in different contexts with objectives related to ensuring access. Box 2 shows the elements that compose each of the identified categories, and it can be noted that several articles appear in more than one category of regulatory technology arrangement.

### Reorganization of services

The arrangements categorized as “Reorganization of services” involve in their organizational/operational nature essentially work processes and are situated strongly in the micropolitical sphere, with a predominance of the use of light technologies ^32^.

We identified 32 articles, representing 71% of the total results. Thus, due to the scope of this category, it is possible to observe variety in the target population, location of the study and types of service reorganization arrangements. Regarding the target population, most studies (40%) refer to services for the treatment of COVID-19, for patients with either an unconfirmed or confirmed diagnosis ^2^ ^,^ ^5^ ^,^ ^33^ ^,^ ^34^ ^,^ ^35^ ^,^ ^36^ ^,^ ^37^ ^,^ ^38^. Subsequently, studies related to elective procedures/surgeries represent 18% of the total, and other types of target population were also listed, such as patients in psychiatry, pediatrics, cardiology, vascular surgery, head and neck, among others.

As for the types of regulatory technology arrangement found in the category called “Reorganization of services”, it was possible to group the experiences into 6 subcategories: (a) implementation of flows/protocols, differentiated between triage, admission, transfer, patient separation and internal protocols; (b) change in supply, including reductions, expansions and readjustments to maintain the supply; (c) readjustment in the work process, distributed among training, reallocation of professionals, change in care procedures and collection of epidemiological data; (d) structural readjustment; identified as restructuring of physical spaces, separation of wards and change in the use of certain environments to meet the demand; (e) structuring of a transfer/regulation center, allocated between the implementation of a transfer center, centralization of the management of the center, and establishment of a specific referral system to meet the needs imposed by the pandemic; and (f) request for private beds.

It was evident that certain arrangements, such as the implementation of flows and protocols, concentrate a large part of the studies, often as an adaptation in the work processes, in line with other arrangements, such as the expansion of supply. However, it is essential to note that most of the arrangements in this category used existing resources, with a primary focus on work processes or the readjustment of the purpose of existing services or wards.

### Use of virtual tools and artificial intelligence

The arrangements that compose the category called “Use of virtual tools and artificial intelligence” are similar due to the use of technology as a watershed in the bed regulation process, although the articles contained in this category also emphasize the need to reorganize the services and new regulatory technology arrangements, with the highlight here being the use of technology itself.

This category represents 44% of the articles included, totaling 20 articles, and its main target population consists of patients with unconfirmed or confirmed COVID-19 diagnosis (69%). Studies related to diabetes patients, pregnant women, orthopedic surgery patients and dialysis patients were also identified.

We can note in this category the existence of subcategories, predominantly grouped by the type of technology used: (a) triage/regulation tool to support clinical decision-making using artificial intelligence and information and communication technology (ICT), identified among the use of applications to support admission or discharge decision-making, use of algorithms to predict the patient severity/length of hospital stay, and artificial intelligence for patient triage and follow-up; (b) use of telemedicine, having been used in the different stages of care (admission, monitoring and discharge), including preoperative assessment and remote matrix support between hospitals; (c) remote patient follow-up to monitor the evolution of the clinical condition, using artificial intelligence in the process of monitoring / indication of hospitalization and in the implementation of self-examination as part of the follow-up process; and (d) creation of a virtual ward, enabling remote follow-up of the evolution of certain patients.

The review of the articles highlighted the use of technology-mediated regulatory technology arrangements prior to the pandemic, which at the time of the health emergency underwent adaptations, including the incorporation of technological tools in processes implemented in the period due to the situation.

### Creation of alternative spaces

Finally, the category called “Creation of alternative spaces”, which includes articles that address the creation of differentiated physical structures for the care of patients during the pandemic and highlights the role of elements called “hard technologies”; however, the full reading of the studies shows micropolitical dimensions and the use of light and light-hard technologies ^32^ as an instrument for managing the physical structures created, enabling the production of the regulatory response in the health crisis.

The literature survey showed the creation of alternative spaces used during the pandemic as an alternative to expand existing installed capacity, through the construction of field hospitals, use of extension spaces connected to hospitals and other services, and creation of community structures, among other arrangements. The structures found in this category were not only at the service of the care of COVID-19 patients, but also of the others, enabling the separation of demand and continuity of care.

In addition, it is possible to identify the use of hotels as structures adapted for “hospital” purposes, aiming at the accomodation of cases with lower severity and separation of patients, with their importance associated with the expansion of the supply and prioritization of the use of hospital beds for cases with higher severity. The share of this type of arrangement in the included publications was relatively limited, including about 6 articles, and most of the studies focused on the target population related to COVID-19, composed of individuals with unconfirmed or confirmed diagnosis. The studies included in this category also contributed significantly in terms of results. Other aspects were also identified with the implementation of the structures, such as a reduction in the need for hospitalization ^39^ ^,^ ^40^, a reduction in the length of hospital stay ^41^, operational maintenance of care not related to COVID-19 ^42^ and the use of these structures as an important source of revenue for hotels ^43^.

## Discussion

As we explore the results of this study, we observe the emergence of a complex and multifaceted context, which sheds light on the production of regulatory technology arrangements amid the pandemic and the possibilities of expanding access and reorganizing health care systems. This overview raises crucial questions regarding the importance of regulatory technology arrangements to address the pandemic, the challenges in relation to their continuity and a look into equity, based on the concept of access regulation that underlie the study.

The issues explored by this review, which addresses the use of regulatory technology arrangements during the pandemic, showed the importance of implementing these mechanisms as a means to respond to the urgency of the health crisis, as well as their effectiveness in expanding access to health care services in a context marked by uncertainties and the rapid increase in the number of cases. From the perspective of demand management, considerable elements are the decrease in the hospitalization or emergency service demand through the redirectioning of demand ^6^ ^,^ ^41^ ^,^ ^44^ ^,^ ^45^ ^,^ ^46^ ^,^ ^47^ ^,^ ^48^ and the reduction in length of hospital stay ^41^ ^,^ ^49^ ^,^ ^50^ as a result of the implementation of regulatory technology arrangements, using remote follow-up, artificial intelligence, implementation of specific flows and protocols in effective demand management.

Regarding the supply, although it was demonstrated that there was a reduction in elective and non-urgent procedures ^14^ ^,^ ^25^ ^,^ ^34^ ^,^ ^35^ ^,^ ^51^ ^,^ ^52^ ^,^ ^53^, as well as an increase in the waiting time for certain procedures ^51^, several of the implemented regulatory technology arrangements contributed to the maintenance of other health care actions not related to COVID-19 ^42^ ^,^ ^49^ ^,^ ^54^ ^,^ ^55^ ^,^ ^56^ ^,^ ^57^ ^,^ ^58^, with increased effectiveness ^53^, optimized supply ^59^ ^,^ ^60^ and increased number of beds ^2^ ^,^ ^14^ ^,^ ^34^.

Field hospitals constituted another important element, as they were another device in the construction of the response and can be considered as one of the regulatory technology arrangements widely used at the time of the pandemic, as indicated in the literature ^61^ ^,^ ^62^ ^,^ ^63^ ^,^ ^64^ ^,^ ^65^. They can be defined as a temporary medical care facilities that can be rapidly configured to respond to emergencies or disasters. In addition, experience reports indicate the importance of these structures in peak transmission periods, with important considerations regarding the difficulties faced in operation, considering the scarcity of material and human resources. However, although this can be considered a relevant regulatory technology arrangement, it was not identified through the search engine used in the construction of this review, which may reinforce the lack of uniformity of the descriptors mentioned above.

Some studies have also reported that the implementation of bed management and transfer centers using digital health technology can be considered an essential regulatory technology arrangement for improving supply and demand management during the pandemic ^5^ ^,^ ^33^ ^,^ ^36^ ^,^ ^52^ ^,^ ^66^. However, it is essential to note that most of the arrangements developed through the use of technology during the pandemic had some type of structure that was used prior to the rise of the pandemic, which facilitated the adaptation and expedited the readjustment process in several cases.

In relation to regulatory technology arrangements based on the implementation of new work processes and the reorganization of the services, the existing organizational structure and the processes and procedures implemented were used as a starting point for the development of new flows, protocols and actions aimed at expanding access ^14^ ^,^ ^25^ ^,^ ^40^ ^,^ ^43^ ^,^ ^51^ ^,^ ^52^ ^,^ ^56^ ^,^ ^59^ ^,^ ^67^. This regulatory technology arrangement category is related to the concept of care-producing regulation, using light and relational technologies, promoting more fluid communication within the services and establishing experiences of living care “in action” ^22^ ^,^ ^23^ ^,^ ^68^.

Some measures, such as the designation of specific teams, in a context that required crisis management in the establishment of local and regional coordination ^5^ ^,^ ^33^ ^,^ ^66^, the implementation of patient regulation and transfer centers ^5^ ^,^ ^36^ ^,^ ^52^ ^,^ ^59^, including the appointment of committees and coordinators, demonstrate a trend in the centralization of processes, which expedite modifications and standardize actions, being recognized as beneficial for facing the health crisis.

The results shown here indicate a number of essential factors that contributed significantly to the successful adaptation of health care services during the pandemic. Among these, we note the flexible organization of services, streamlined decision-making, improved communication between health care providers, and the rise of a culture of solidarity and empathy.

The adaptation of regulatory technology arrangements during the pandemic provided valuable lessons; however, transforming strategies into something lasting will require a continuous effort and a focus on regulation, financing and equity, aiming to build more resilient health care systems capable of responding effectively to future challenges. The different governance models used globally also pose an important challenge in consolidating arrangements in a more regionalized and articulated manner within existing health care systems.

Although there is no single solution that applies to all circumstances, the identification of the elements that contributed to the process can be an important legacy of the pandemic; however, to advance in this reflection it is necessary to ask: to what extent can we continue to innovate when implementing dynamic regulatory technology arrangements, based on streamlined regulation that connects health care services, including providers and managers, to the needs of users?

Among the limitations of this study, we note insufficient information, in primary studies, on the developments and continuity of the implemented regulatory technology arrangements. Although some authors have mentioned the importance of the arrangements implemented for facing new health challenges, as well as for the daily routine of services ^53^ ^,^ ^54^ ^,^ ^69^, it is not possible to determine which of these arrangements remain in operation and the effects produced. A good example is telehealth, which saw a significantly increased use during the pandemic ^7^; however, as the pandemic declined, it had a decrease in use levels ^44^.

Another limitation of the study concerns the diversity of health care systems included in this review. Based on fundamental characteristics of the composition of health care systems such as the type of coverage, the exercise of governance and financing, and the look into use and access, the differences found must be considered in a more in-depth analysis of the responses produced globally.

## Conclusion

The pressure on health care systems caused by the severity and very high lethality of COVID-19 contributed to show and intensify the urgency and the need for readiness for action. This situation favored the implementation of new regulatory technology arrangements, which also had some degree of relaxation of bureaucratic rules and procedures, as well as greater administrative autonomy.

It should be noted that, for the most consolidated health care systems, the resilience capacity - using resources developed before the COVID-19 pandemic - was greater and enabled not only the development of a better response, but the ability to develop existing instruments and processes for a new challenging situation.

However, although the new arrangements used to address the crisis showed positive results such as expanded supply, streamlined access, and use of technology to enable care, among others, the maintenance of these regulatory technology arrangements in the daily routine of health care services includes the need to overcome regulatory barriers, establishment of standards for quality, safety and funding in a sustainable manner. For the various existing strategies that underwent adaptations to the pandemic context, it is necessary to address these issues so long-term actions can be planned, when applicable.

In addition, it is necessary to address the lack of uniformity in the definition of the very concept of access regulation in the global context, considering that the fragmentation of health care systems that hinders emergency response is a real issue that requires urgent addressing to achieve the goal of making health care systems increasingly resilient. On the other hand, the power of access regulation as a tool for the production of health care should be recognized, taking into account the strength of micropolitical processes in health care and the centrality of users in the production of care, even considering the existing hegemonic medical model, constituting an important field of dispute.

Also, the matter of equity cannot be neglected, although these innovations have shown their value, it is essential to ensure that the improvement in access to health care services is equitable, especially when considering the plurality of communities and population groups in their different territories, equipped with very particular cultural constructions. Considering the multifaceted micropolitical core of these processes is fundamental in combating the inequities in access to health care services observed in systems of countries strongly marked by social inequality, as is the case of countries located in the Global South.

It is essential to conduct a more in-depth identification and assessment of arrangements and lessons learned during the COVID-19 pandemic that have been established in a more consolidated manner in health care systems in the post-pandemic context, since this assessment will support analyses for other complex health situations to come in the future.
