# Supplementary material for: Regulatory technology arrangements for hospital bed access during the COVID-19 pandemic: scope review
Source: Cad Saude Publica. 2025 Jun 27;41(5):e00044824. doi: 10.1590/0102-311XEN044824 (PMC12212299; doi:10.1590/0102-311XEN044824)
Supplement: Translation

# Translation: Arranjos tecnológicos de regulação de acesso à leitos hospitalares no enfrentamento da pandemia de COVID-19: revisão de escopo

**DOI:** 10.1590/0102-311XPT044824

**Authors:** Mariana Prado Freire, Arthur Chioro, Fernando Tureck, Luís Fernando Nogueira Tofani, Letícia Gabriela da Silva, André Luiz Bigal, Amanda da Cruz Santos Vieira, Marília Cristina Prado Louvison

**Affiliations:** 1 Faculdade de Saúde Pública, Universidade de São Paulo, São Paulo, Brasil.; 2 Universidade Federal de São Paulo, São Paulo, Brasil.; 3 Universidade Metropolitana de Santos, Santos, Brasil.; 4 Universidade do Contestado, Mafra, Brasil.; 5 Faculdade Santa Marcelina, São Paulo, Brasil.

**Keywords:** Regulação e Fiscalização em Saúde, Acesso aos Serviços de Saúde, COVID-19, Leitos Hospitalares

## Abstract

A pandemia de COVID-19 impôs inúmeros desafios aos sistemas de saúde, incluindo o acesso a leitos hospitalares. Analisar a resposta ofertada e identificar arranjos tecnológicos de regulação que contribuíram no acesso ao cuidado pode cooperar na preparação dos sistemas para emergências futuras e ainda oferecer alternativas para dificuldades atuais. O objetivo deste estudo é mapear, sintetizar e categorizar os arranjos tecnológicos de regulação utilizados no acesso aos leitos hospitalares no contexto do enfrentamento da pandemia de COVID-19 utilizando o método da revisão de escopo. A revisão utilizou as recomendações do Instituto Joanna Briggs e o relatório PRISMA-ScR, além do acrônimo PCC (população [P]: pacientes com ou sem COVID-19; conceito [C]: regulação de acesso a leitos; e contexto [C]: pandemia de COVID-19). A busca foi realizada nas bases PubMed, Scopus, Embase, Web of Science e LILACS entre julho e setembro de 2022, e foram selecionados 45 artigos para a revisão. Foram estabelecidas três categorias de análise: Reorganização de serviços; Uso de ferramentas virtuais e de inteligência artificial; e Criação de espaços alternativos, que auxiliaram na identificação e análise dos arranjos tecnológicos de regulação utilizados. Destaque para arranjos como a telessaúde e telemedicina, a reconfiguração de espaços existentes para a ampliação da capacidade de assistência e modificações no processo de trabalho, incluindo a adoção de protocolos específicos e estabelecimento de fluxos diferenciados. A revisão evidenciou a importância de arranjos tecnológicos de regulação no acesso a leitos e desafios como a regulamentação e o financiamento, fatores que podem ser fundamentais para o uso de arranjos tecnológicos de regulação.

## Introdução

Devido à crise global de saúde ocasionada pelo SARS-CoV-2, um tipo de coronavírus, em 30 de janeiro de 2020, a Organização Mundial da Saúde (OMS) declarou Emergência de Saúde Pública de Importância Internacional (ESPII). Para coordenar a resposta à nova emergência sanitária, lançou um conjunto de recomendações englobando desde o apoio aos sistemas de saúde vulneráveis até o desenvolvimento acelerado de vacinas e terapias ^1^.

Paralelamente à declaração da ESPII e do reconhecimento da pandemia pela OMS, em 11 de março de 2020, à medida que novos casos e óbitos decorrentes da COVID-19 eram registrados, suscitaram reações diversas e por vezes desordenadas de resposta à saúde em todo o mundo. Evidências apontam que a identificação das necessidades e a organização da resposta à pandemia, especialmente durante a fase inicial, basearam-se principalmente no conhecimento adquirido no enfrentamento à outras crises sanitárias significativas e na estrutura de saúde local já existente ^2^ ^,^ ^3^, nesse sentido, a pandemia evidenciou as forças e fraquezas existentes nos sistemas de saúde, resultando indistintamente em respostas variadas e frequentemente questionáveis, mesmo em sistemas de saúde reconhecidos no âmbito internacional ^4^.

Além da OMS, entidades internacionais como o Centro de Controle e Prevenção de Doenças (CDC, acrônimo em inglês) dos Estados Unidos e o Centro Europeu de Prevenção e Controle de Doenças (ECDC, acrônimo em inglês) emitiram recomendações que serviram como base científica e diretriz prática para a implementação de ações, incluindo medidas de vigilância em saúde e gestão dos serviços. Embora as orientações sobre a implantação de medidas não farmacológicas tenham sido protagonizadas durante a pandemia, as estratégias voltadas para a assistência dos pacientes ganharam ênfase, a exemplo do monitoramento de casos por telefone ou aplicativos, expansão da capacidade hospitalar e a implementação de ferramentas de telessaúde ^5^ ^,^ ^6^ ^,^ ^7^.

Os primeiros países atingidos pelo vírus serviram como importantes pontos de referência para a preparação da resposta dos sistemas de saúde à pandemia. A Itália, por exemplo, recomendou treinamento de pessoal, aquisição de equipamentos e insumos, designação de equipes e áreas dedicadas ao atendimento de pacientes com COVID-19 ^8^ ^,^ ^9^ ^,^ ^10^ ^,^ ^11^ ^,^ ^12^ ^,^ ^13^. Entretanto, a observação das recomendações de autoridades internacionais para ampliação de leitos, suspensão de procedimentos eletivos, aumento do efetivo de trabalhadores ^2^ ^,^ ^14^, não foi suficiente para evitar que as demandas por cuidados de saúde se sobrepusessem à capacidade instalada dos serviços.

Alguns países com sistemas de saúde, circunstancialmente sob pressão e alertados para o risco de colapso iminente, instituíram mecanismos regulatórios que foram fundamentais para a organização da resposta, na tentativa de corrigir as lacunas entre oferta e demanda. Nesse sentido, de forma conceitual, a regulação em saúde é o campo da Saúde Pública que atua na mediação entre as necessidades de saúde e a disponibilidade, intervindo nas relações de mercado existentes em busca de equilíbrio entre oferta e demanda ^15^.

Saltman & Busse ^16^ destacam que, na Europa, o conceito de regulação é apontado como um processo não linear construído de maneira a considerar uma variedade de ferramentas regulatórias que se estabeleceram ao longo da construção dos sistemas de saúde. Já no contexto do Brasil, especificamente no âmbito do Sistema Único de Saúde (SUS), a regulação é entendida como uma função de gestão abrangendo desde aspectos estruturais da regulação de sistemas até a regulação de serviços e, finalmente, a regulação do acesso. Esta última é definida como “*a disponibilização da alternativa assistencial mais adequada às necessidades do cidadão, de forma equitativa, organizada, oportuna e qualificada, efetivada por meio de complexos reguladores*” ^17^.

Na prática, as características burocráticas da regulação em saúde no Brasil frequentemente divergem das necessidades cotidianas dos serviços de saúde e dos cidadãos, de acordo com as observações dos envolvidos. As construções políticas e teóricas relacionadas à macrorregulação não se mostram suficientes para garantir o acesso dos usuários aos serviços de saúde e a regulação do acesso apresenta importantes desafios no sentido de efetivamente aproximar a oferta da demanda ^18^.

Nesse sentido, os conceitos mais ampliados de acesso auxiliam na análise dos processos regulatórios, extrapolando a dimensão geográfica ou de disponibilidade, conforme identificado em revisão realizada por Travassos & Martins ^19^. De forma sintética, para as autoras a ideia de acessibilidade de Donabedian, em sua dimensão sócio-organizacional, leva em consideração os fatores que favorecem ou dificultam o acesso, bem como a adequação dos profissionais e recursos tecnológicos na atenção à demanda ^19^. Corroboram, ainda que com divergências conceituais, com as contribuições de Andersen ^20^, oferecendo um olhar para o acesso para além da entrada no serviço de saúde, incluindo o percurso e os cuidados subsequentes necessários.

Um outro refinamento do conceito de acesso é introduzido por Penchansky & Thomaz (1981, *apud* Travassos & Martins ^19^), denominado como acolhimento, que diz respeito ao ajuste entre a oferta e demanda, levando em consideração a forma como os serviços se organizam para receber os pacientes e como estes recebem o cuidado ofertado. A polissemia conceitual de acesso favorece a análise da produção do cuidado adotada durante a pandemia, considerando a variedade de fluxos assistenciais produzidos dentro e fora dos hospitais durante a crise sanitária, incluindo os desafios em relação aos cuidados sucessivos, os processos de tomada de decisão em relação às necessidades e ofertas, além da organização das ações.

De acordo com a perspectiva de Cecílio et al. ^21^, a regulação do acesso cria um cenário de conflito, no qual os atores envolvidos no processo desempenham papéis importantes, indo além do mero “*ordenamento burocrático-administrativo*”, isso envolve a operação em diferentes “*regimes de regulação*”, abrangendo as diferentes dimensões do usuário, trabalhador, gestor, prestador e outros. Nesse sentido, vários autores têm contribuído para reformular a concepção da regulação do acesso como um processo em constante evolução, moldado na micropolítica dos serviços de saúde ^22^ ^,^ ^23^.

Em um contexto mais amplo, quando distanciado das ações agenciadas por processos burocráticos e estruturados, os movimentos de regulação do acesso se ocupam da produção do cuidado na tentativa de superar modelos médico-hegemônicos e abrem espaço para diferentes saberes do campo da Saúde Pública. Esses movimentos, inseridos no campo da micropolítica do trabalho em saúde, valorizam a organização do trabalho “*vivo*” em ato e a força das relações interpessoais na prática em saúde ^24^ e têm sido denominados como arranjos tecnológicos de regulação ^22^ ^,^ ^23^. Em outras palavras, os arranjos tecnológicos de regulação são ferramentas essenciais que contribuem para a organização do cuidado em saúde, permitindo uma visão mais ampla da regulação do acesso além do tradicional complexo regulador, contemplando estratégias inovadoras que atendam às necessidades de saúde da população ^22^.

Durante a pandemia, com demandas acentuadas e ofertas extremamente insuficientes, os papéis centrais da regulação podem ter se deslocado e desencadeado respostas diversas. A identificação dos arranjos tecnológicos de regulação exitosos no período que contribuíram para o acesso a leitos e fizeram frente à demanda extraordinária e ao altíssimo número de óbitos ^25^ pode ser valiosa não apenas para manejo de futuras crises de saúde, mas também para fornecer novos modelos de enfrentamento das dificuldades diárias no campo da regulação. Além disso, a resiliência dos sistemas de saúde foi essencial para adaptar-se rapidamente às circunstâncias adversas, mantendo a capacidade de resposta diante de desafios imprevistos ^26^. Assim, o objetivo deste estudo é mapear, sintetizar e categorizar os arranjos tecnológicos de regulação utilizados no acesso dos leitos hospitalares no contexto do enfrentamento da pandemia de COVID-19, incluindo os desdobramentos na atenção ambulatorial e o cuidado para pacientes não COVID-19, quando relacionados.

## Metodologia

Esta revisão integra a pesquisa financiada pela Fundação de Amparo à Pesquisa do Estado de São Paulo (FAPESP) com recursos do Programa de Pesquisa para o SUS (PPSUSCovid), intitulada *Enfrentamento da Pandemia de COVID-19: Produções, Invenções e Desafios na Gestão do Cuidado em Rede*. A necessidade de fazer uma revisão sobre a temática foi identificada durante a realização da pesquisa, com o objetivo de mapear a produção acadêmica existente sobre o tema, de forma abrangente, permitindo observar a realidade como base para a compreensão dos achados de pesquisa.

Nesse sentido, a revisão de escopo foi escolhida pela aptidão para evidenciar conceitos e definições e identificar fatores relacionados ao conceito ^27^. A construção metodológica foi realizada de acordo com as recomendações do Instituto Joanna Briggs para revisões de escopo e pelo relatório *Preferred Reporting Items for Systematic Reviews and Meta-Analyses: Extension for Scoping Reviews* (PRISMA-ScR; Itens de Relatórios Preferenciais para Revisões Sistemáticas e Metanálises: Extensão para Revisões de Escopo), incluindo suas atualizações ^28^ ^,^ ^29^.

Alinhada com o objeto de pesquisa principal, que se debruçou sobre a identificação de arranjos e inovações no enfrentamento da pandemia, a pergunta de pesquisa foi construída na mesma direção, sendo: “Quais arranjos tecnológicos de regulação de acesso a leitos foram produzidos durante a pandemia?”. Após a definição da pergunta de pesquisa, a estratégia de busca foi construída com base no acrônimo PCC (população, conceito, contexto), onde a população (P) inclui pacientes com COVID-19 e sem COVID-19, o conceito (C) se refere à regulação de acesso a leitos e o contexto (C) abrange a pandemia de COVID-19.

A estratégia de busca foi desenvolvida com base nos descritores das plataformas MeSH (https://www.ncbi.nlm.nih.gov/mesh/) e DeCS (https://decs.bvsalud.org) para englobar um espectro abrangente de estudos. Os termos de busca utilizados foram “*covid-19*” *AND* “*acesso à saúde*” *AND* “*regulação em saúde*”, em português e “*covid19*” *AND* “*delivery of healthcare*” *AND* “*regulation in health*”, em inglês. Considerando a particularidade da aplicação do termo “*regulação em saúde*” em português, mas especificamente na literatura brasileira, foram necessárias etapas de calibração, utilizando termos indexados com combinações distintas nas principais bases de dados para verificar a capacidade deles em exprimir a realidade pesquisada.

Após a definição dos termos, as buscas foram realizadas nas bases de dados PubMed, Scopus, Embase, Web of Science e LILACS, a escolha dessas bases foi feita para garantir a cobertura das revistas indexadas no tema e ocorreram nos meses de julho a setembro de 2022, compreendendo os anos de 2020 a 2022, no contexto da pandemia de COVID-19.

Utilizamos a plataforma Rayyan (https://www.rayyan.ai/) para a triagem ^30^, na qual analisamos títulos e resumos, aplicando os critérios de elegibilidade estabelecidos com base no acrônimo PCC. Foram excluídos os registros que não estavam diretamente relacionados ao enfrentamento da pandemia, que não abordavam o acesso a leitos hospitalares ou de urgência, bem como aqueles de natureza opinativa ou prescritiva. A revisão considerou apenas estudos que descreviam os arranjos tecnológicos de regulação implementados durante a pandemia para o atendimento de pacientes com e sem COVID-19, revisões com a mesma temática e não aplicou limitações geográficas. Além disso foram excluídos registros disponíveis em idiomas que não fossem português, espanhol e inglês.

Os registros considerados elegíveis foram reinseridos na plataforma Rayyan e avaliados na íntegra pelos revisores, utilizando os critérios de exclusão estabelecidos, além disso, foi construída uma matriz de extração de dados contendo as informações relevantes a serem coletadas nos registros, incluindo dados de identificação (local, ano, idioma, autores, título, revista) e dados analíticos (tipo de estudo, objetivos, arranjos identificados, população-alvo e resultados alcançados com a implementação do arranjo, quando descritos).

Os resultados foram analisados e sistematizados utilizando técnicas de análise de conteúdo ^31^, partindo de categorias pré-estabelecidas de análise. Após exaustiva manipulação e codificação do material, houve o refinamento das categorias existentes e a criação de subcategorias de análise que possibilitaram o agrupamento e a identificação de conteúdos relacionados aos temas mais relevantes encontrados sobre o assunto na literatura.

A equipe de pesquisadores realizou 13 reuniões online, utilizando a plataforma Google Meet (https://meet.google.com), onde foram discutidas todas as etapas da revisão: concepção, identificação de termos de busca, alinhamento de critérios de inclusão e exclusão, extração de dados e análise. Os encontros virtuais também foram utilizados para a análise coletiva de determinados registros para produção de consenso.

## Resultados

A busca nas bases de dados resultou em 3.713 registros, dos quais 109 eram duplicatas e foram eliminadas, restando 3.604 registros para a primeira etapa. Foram considerados elegíveis 281 registros para leitura completa e aplicação dos critérios de exclusão estabelecidos, utilizando a plataforma Rayyan. A maior parte dos registros excluídos não preencheu os critérios relacionados ao conceito de regulação de acesso a leitos hospitalares (60,2%), seguidos por estudos fora do escopo de publicação (24,8%), fora do cenário da pesquisa (12,2%), fora do contexto da pandemia (1,5%) e em idioma não incluído (1,2%). Após a aplicação rigorosa dos critérios de exclusão, 45 registros foram incluídos na revisão, conforme demonstrado na Figura 1.

**Figura 1:** Fluxograma de identificação, seleção e inclusão de estudos.

Os artigos incluídos, detalhados no Quadro 1, encontram-se distribuídos geograficamente, com predomínio de estudos realizados nos Estados Unidos, representando 37,7% (17 artigos); na sequência, a produção literária europeia, representando 35,5% do total (16 artigos), com destaque para a italiana (7 artigos). Foram incluídos 9 artigos do continente asiático, representando 20% do total, sendo que a Coreia do Sul contribuiu com um terço do total de artigos; além disso, houve 1 artigo brasileiro e 1 australiano, bem como uma revisão de literatura que tem abrangência global, entretanto, 95% dos artigos encontrados são de língua inglesa.

**Quadro 1:** Estudos incluídos na revisão.

ESTUDO	TÍTULO	IDIOMA	PAÍS	ANO DE PUBLICAÇÃO	TIPO DE ESTUDO
Fuchs et al. ^41^	*Assessment of a Hotel-Based COVID-19 Isolation and Quarantine Strategy for Persons Experiencing Homelessness*	Inglês	Estados Unidos	2021	Coorte retrospectiva
Bruni et al. ^43^	*Telemedicine-Enabled Accelerated Discharge of Patients Hospitalized with COVID-19 to Isolation in Repurposed Hotel Rooms*	Inglês	Itália	2020	Relato de experiência
Kang et al. ^39^	*Operating Protocols of a Community Treatment Center for Isolation of Patients with Coronavirus Disease, South Korea*	Inglês	Coreia do Sul	2020	Estudo descritivo
Paganini et al. ^40^	*Implementation of a nurse-led alternate care site for the management of the surge of patients with COVID-19 in an Italian emergency department*	Inglês	Itália	2022	Estudo retrospectivo
Bar-On et al. ^42^	*Establishing a COVID-19 Treatment Centre in Israel at the Initial Stage of the Outbreak: Challenges, Responses and Lessons Learned*	Inglês	Israel	2021	Estudo retrospectivo
Lee et al. ^59^	*COVID-19 Level-Loading: Transferring Emergency Department Patients to a Partner Academic Medical Center Within a Healthcare System*	Inglês	Estados Unidos	2021	Relato de experiência
Lowe et al. ^33^	*Avoiding Crisis Conditions in the Healthcare Infrastructure: 2 Case Studies in Statewide Collaboration*	Inglês	Estados Unidos	2022	Estudo de casos múltiplos
Ranzani Rigotti et al. ^34^	*Resilience of Healthcare Systems in the Face of COVID-19: An Experience Report*	Português/Inglês	Brasil	2022	Relato de experiência
Longo et al. ^54^	*Managing Head and Neck Cancer Patients During the COVID-19 Pandemic: The Experience of a Tertiary Referral Center in Southern Italy*	Inglês	Itália	2021	Relato de experiência
Shrestha et al. ^35^	*Anesthesiology and Critical Care Response to COVID-19 in Resource-Limited Settings: Experiences from Nepal*	Inglês	Nepal	2021	Relato de experiência
Al Garhy et al. ^70^	*Mental Health Services During the COVID-19 Pandemic in Abu Dhabi, UAE*	Inglês	Emirados Árabes Unidos	2021	Estudo de caso
O’Rielly et al. ^51^	*Surgery and COVID-19: A Rapid Scoping Review of the Impact of the First Wave of COVID-19 on Surgical Services*	Inglês	Global	2021	Revisão de escopo
Di Bidino & Cicchetti ^25^	*Impact of SARS-CoV-2 on Provided Healthcare. Evidence from the Emergency Phase in Italy*	Inglês	Itália	2020	Revisão realista da literatura
Burden et al. ^55^	*The Provision of a Time-Critical Elective Surgical Service During the COVID-19 Crisis: A UK Experience*	Inglês	Inglaterra	2021	Relato de experiência
Bunch et al. ^56^	*Immuno-Thrombotic Complications of COVID-19: Implications for Timing of Surgery and Anticoagulation*	Inglês	Estados Unidos	2022	Relato de experiência
Mathews et al. ^14^	*Variation in Initial U.S. Hospital Responses to the Coronavirus Disease 2019 Pandemic*	Inglês	Estados Unidos	2021	Estudo observacional
Lee et al. ^67^	*Clinical Pathway for Emergency Brain Surgery During COVID-19 Pandemic and its Impact on Clinical Outcomes*	Inglês	Coreia do Sul	2021	Estudo retrospectivo
Lefrant et al. ^2^	*A National Healthcare Response to Intensive Care Bed Requirements During the COVID-19 Outbreak in France*	Inglês	França	2020	Estudo observacional multicêntrico retrospectivo
Burgansky et al. ^52^	*COVID-19 in a Community Hospital*	Inglês	Estados Unidos	2020	Descritivo
Archer et al. ^66^	*Prioritizing Critical-Care Resources in Response to COVID-19: Lessons from the Development of Thailand’s Triage Protocol*	Inglês	Tailândia	2020	Protocolo
Gagliano et al. ^5^	*COVID-19 Epidemic in the Middle Province of Northern Italy: Impact, Logistics, and Strategy in the First Line Hospital*	Inglês	Itália	2020	Estudo de caso
Giorgi et al. ^49^	*The Management of Emergency Spinal Surgery During the COVID-19 Pandemic in Italy*	Inglês	Itália	2020	Relato de experiência
Samofalov et al. ^36^	*Information And Communication Technologies In Public Management of the Healthcare Institutions Network During COVID-19 Pandemics*	Inglês	Polônia	2020	Estudo descritivo
Settelmeier et al. ^71^	*Capacity Changes in German Certified Chest Pain Units During COVID-19 Outbreak Response*	Inglês	Alemanha	2020	Estudo observacional
Oakley et al. ^37^	*Assembly Line ICU: What the Long Shops Taught Us about Managing Surge Capacity for COVID-19*	Inglês	Inglaterra	2020	Estudo ecológico
Piazza et al. ^57^	*The Challenge of Maintaining Necessary Vascular and Endovascular Services at a Referral Center in Northern Italy During the COVID-19 Outbreak*	Inglês	Itália	2021	Estudo retrospectivo ecológico
Fraymovich et al. ^72^	*A Blueprint for Pediatric Emergency Resource Reallocation During the COVID-19 Pandemic: An NYC Hospital Experience*	Inglês	Estados Unidos	2020	Estudo retrospectivo ecológico
Hartford et al. ^60^	*Pediatric Emergency Department Responses to COVID-19: Transitioning from Surge Preparation to Regional Support*	Inglês	Estados Unidos	2020	Estudo descritivo
Cuschieri et al. ^58^	*The COVID-19 Pandemic: Lessons Learned for Sustained Trauma Preparedness and Responses*	Inglês	Estados Unidos	2021	Estudo descritivo
Wood et al. ^38^	*The Value of Triage During Periods of Intense COVID-19 Demand: Simulation Modeling Study*	Inglês	Reino Unido	2021	Descritivo/Estudo de caso
Hinson et al. ^73^	*Multisite Implementation of a Workflow-Integrated Machine Learning System to Optimize COVID-19 Hospital Admission Decisions*	Inglês	Estados Unidos	2022	Relato de experiência
Blazey-Martin et al. ^74^	*Primary Care Population Management for COVID-19 Patients*	Inglês	Estados Unidos	2020	Avaliação de implementação
Kesavadev et al. ^44^	*A New Interventional Home Care Model for COVID Management: Virtual Covid IP*	Inglês	Índia	2021	Relato de experiência
Bircher et al. ^75^	*Remote Care and Triage of Obstetric Patients with COVID-19 in the Community: Operational Considerations*	Inglês	Reino Unido	2022	Relato de experiência
Panagopoulos et al. ^45^	*Intelligent Pervasive Monitoring Solution of COVID-19 Patients*	Inglês	Grécia	2022	Relato de experiência
Greven et al. ^76^	*Telemedicine in Spine Surgery: Outcomes for 138 Patients with Virtual Preoperative Assessment Compared to Historical Controls*	Inglês	Estados Unidos	2022	Estudo de caso-controle
Singh et al. ^69^	*Impact of Telemedicine on Hospitalisation and Mortality Rates in Community-Based Haemodialysis Centres in Singapore During the COVID-19 Pandemic*	Inglês	Singapura	2020	Estudo de coorte
Kim et al. ^46^	*A Brief Telephone Severity Scoring System and Therapeutic Living Centers Solved Acute Hospital-Bed Shortage During the COVID-19 Outbreak in Daegu, Korea*	Inglês	Coreia do Sul	2020	Estudo descritivo
Koziatek et al. ^6^	*Assessing the Impact of a Rapidly Scaled Virtual Urgent Care in New York City During the COVID-19 Pandemic*	Inglês	Estados Unidos	2020	Coorte retrospectiva
Carlberg et al. ^77^	*Preliminary Assessment of a Telehealth Approach to Evaluating, Treating, and Discharging Low-Acuity Patients with Suspected COVID-19*	Inglês	Estados Unidos	2020	Estudo retrospectivo - tipo coorte
Ferry et al. ^47^	*A Virtual Ward Model of Care for Patients With COVID-19: Retrospective Single-Center Clinical Study*	Inglês	Austrália	2021	Estudo retrospectivo - tipo coorte
Borgen et al. ^50^	*From Hospital to Home: An Intensive Transitional Care Management Intervention for Patients with COVID-19*	Inglês	Estados Unidos	2020	Estudo observacional - tipo coorte
Kodama et al. ^48^	*Reengineering the Discharge Transition Process of COVID-19 Patients Using Telemedicine, Remote Patient Monitoring, and Around-the-Clock Remote Patient Monitoring from the Emergency Department and Inpatient Units*	Inglês	Estados Unidos	2020	Estudo retrospectivo
Fuentes et al. ^53^	*Impacto de la Pandemia de COVID-19 en la Organización Asistencial del Ictus. Plan Ictus Madrid*	Espanhol	Espanha	2020	Avaliativo
Lai et al. ^78^	*Digital Triage: Novel Strategies for Population Health Management in Response to the COVID-19 Pandemic*	Inglês	Estados Unidos	2020	Descritivo

Fonte: elaboração própria.

Em relação ao ano de publicação, observamos uma maior concentração de artigos publicados em 2020, primeiro ano da pandemia (22 artigos). Em 2021, identificamos 15 artigos, com redução proporcional em 2022, último ano analisado (8 artigos). Dentre os estudos identificados, houve uma grande parcela de relatos de experiência (11 artigos), estudos retrospectivos (9 artigos), estudos descritivos (7 artigos) e estudos de casos (4 artigos).

Durante o processo de extração de dados dos artigos incluídos, a identificação de arranjos tecnológicos de regulação utilizados no acesso aos leitos hospitalares foi priorizada, uma vez que esta informação é crucial para a resposta da pergunta de pesquisa, também foram extraídos os objetivos da ação e resultados obtidos mediante a aplicação do arranjo descrito.

A partir da análise em primeiro plano, que inclui o detalhamento dos arranjos tecnológicos de regulação utilizados, a população-alvo, objetivo e resultados, três categorias de análise foram estabelecidas: (i) Reorganização de serviços, tópico que abrange arranjos diretamente relacionados a gestão e força de trabalho; (ii) Uso de ferramentas virtuais e de inteligência artificial, destacando o uso de tecnologia na implantação dos arranjos; e (iii) Criação de espaços alternativos, prioritariamente relacionados a questões que envolveram infraestrutura e ampliação/modificação de espaços físicos, conforme demonstrado no Quadro 2. Cada uma das categorias consiste em um agrupamento dos arranjos tecnológicos de regulação para acesso aos leitos durante a pandemia de COVID-19 que guarda relação entre os aspectos citados anteriormente, sendo a natureza dos arranjos o elemento principal de sua categorização. É possível observar também arranjos tecnológicos de regulação que se produziram em ações de atenção ambulatorial, inclusive para casos não COVID-19, porém estas se apresentaram como desdobramentos ou consequências das ações e arranjos voltados para atenção hospitalar e o acesso à leitos, gerando redução de oferta ambulatorial em determinadas situações.

**Quadro 2:** Categorias de análise e subcategorias.

CATEGORIA DE ANÁLISE	SUBCATEGORIA	DESCRIÇÃO
Reorganização de serviços	1.1) Implantação de fluxos/protocolos	Implantação de protocolo de triagem ^5^ ^,^ ^14^ ^,^ ^36^ ^,^ ^40^ ^,^ ^43^ ^,^ ^51^ ^,^ ^56^ ^,^ ^67^
		Implantação de protocolo de admissão/protocolo de acesso/protocolo de COVID-19 ^25^ ^,^ ^40^ ^,^ ^51^ ^,^ ^52^ ^,^ ^54^ ^,^ ^57^ ^,^ ^60^ ^,^ ^66^ ^,^ ^70^
		Implantação de protocolo de transferência de pacientes ^33^
		Implantação de protocolos internos (segregação de pacientes) ^35^ ^,^ ^39^ ^,^ ^49^
		Alteração de fluxos internos do serviço ^2^ ^,^ ^34^ ^,^ ^36^ ^,^ ^37^ ^,^ ^55^ ^,^ ^58^ ^,^ ^72^
	1.2) Alteração da oferta	Aumento da oferta (para atendimento de COVID-19) ^2^ ^,^ ^5^ ^,^ ^14^ ^,^ ^33^ ^,^ ^35^ ^,^ ^37^ ^,^ ^39^ ^,^ ^40^
		Redução da oferta - atendimentos ambulatoriais, eletivos e não urgentes - poupando recursos humanos, materiais e espaço físico para o atendimento de COVID-19 ^2^ ^,^ ^5^ ^,^ ^25^ ^,^ ^35^ ^,^ ^51^ ^,^ ^52^ ^,^ ^53^
		Reorganização do serviço para a manutenção da oferta de procedimentos eletivos ^42^ ^,^ ^49^ ^,^ ^54^ ^,^ ^55^ ^,^ ^56^ ^,^ ^57^ ^,^ ^58^
	1.3) Readequação no processo de trabalho	Treinamento, implantação de novas práticas clínicas ^14^ ^,^ ^36^ ^,^ ^37^ ^,^ ^48^ ^,^ ^50^ ^,^ ^51^ ^,^ ^52^ ^,^ ^60^
		Realocação de profissionais ^2^ ^,^ ^53^ ^,^ ^70^
		Readequação nos processos de logística interna ^25^ ^,^ ^51^
		Alteração no modelo de triagem (“triagem reversa”) ^43^
		Mudança na coleta de dados epidemiológicos para auxiliar no planejamento ^5^
	1.4) Readequação estrutural	Reestruturação do espaço físico do serviço ^5^ ^,^ ^53^ ^,^ ^58^ ^,^ ^60^ ^,^ ^71^ ^,^ ^72^
		Separação de alas ^39^ ^,^ ^42^
		Mudança na destinação do espaço físico para ampliação da oferta - transformação de salas de recuperação anestésica, ala de agudos em espaços destinados ao atendimento de pacientes com COVID-19 ^2^ ^,^ ^14^ ^,^ ^35^ ^,^ ^70^
	1.5) Estruturação de central de transferência/regulação	Implantação de central de transferência para coordenar o fluxo de pacientes entre serviços ^14^ ^,^ ^33^ ^,^ ^59^
		Nomeação de coordenador responsável pela central de transferências - centralizando as responsabilidades e articulação de rede ^59^
		Estabelecimento de sistema de referência com hospitais do entorno ^39^
	1.6) Requisição de leitos privados	Requisição de leitos privados, a partir de estabelecimento de normativas construídas com articulação política dos atores ^33^
Uso de ferramentas virtuais e de inteligência artificial	2.1) Ferramenta de triagem/regulação para apoio à decisão clínica - uso de inteligência artificial e tecnologias da informação e comunicação	Emprego da tecnologia para decisão de admissão/alta de pacientes ^6^ ^,^ ^33^ ^,^ ^73^
		Desenvolvimento e utilização de algoritmo para previsão de gravidade dos casos ^74^ ^,^ ^75^
		Uso de inteligência artificial na triagem e monitoramento remoto de pacientes ^48^ ^,^ ^78^
	2.2) Uso de telemedicina telessaúde	Utilização para tele para admissão, acompanhamento e alta dos pacientes - “transição do cuidado” para casa ^36^ ^,^ ^42^ ^,^ ^48^ ^,^ ^50^ ^,^ ^53^ ^,^ ^54^ ^,^ ^69^ ^,^ ^77^
		Implantação de telematriciamento entre hospitais na gestão de casos ^36^
		Uso da telemedicina na avaliação pré-operatória ^76^
	2.3) Monitoramento remoto de pacientes	Monitoramento remoto de pacientes para acompanhar a evolução do quadro clínico e critérios de internação ^36^ ^,^ ^44^ ^,^ ^46^ ^,^ ^47^ ^,^ ^48^ ^,^ ^50^ ^,^ ^75^
		Utilização de algoritmo/inteligência artificial no monitoramento de pacientes ^45^ ^,^ ^47^ ^,^ ^74^
		Implantação de autoexame ^40^
	2.4) Criação de ala virtual	Implantação de ala virtual, inserindo o paciente em programa de monitoramento remoto após avaliação e classificação de risco ^47^ ^,^ ^50^ ^,^ ^75^
Criação de espaços alternativos	3.1) Implantação de estrutura comunitária/alternativa - “Hospitais de Campanha”	Implantação de estrutura comunitária para acompanhamento de pacientes sem sinal de gravidade ^39^
		Criação de espaço alternativo para atendimento de pacientes com COVID-19, baseados na demanda da região ^33^
		Criação de espaço alternativo liderado por enfermeiras ^40^
		Implantação de estrutura anexa ao hospital para atendimento de pacientes com COVID-19, promovendo separação total do espaço garantindo continuidade ao atendimento não-COVID-19 ^42^
	3.2) Implantação de leitos hospitalares para pacientes com COVID-19 em hotéis com retaguarda de profissional de saúde	Uso dos leitos para o isolamento de pacientes com COVID-19, em situação de rua ^41^
		Uso dos leitos para acelerar a alta hospitalar, dando continuidade ao tratamento no leito implantado em hotel ^43^

Fonte: elaboração própria.

Os achados nesta revisão de literatura demonstram a relevância dos arranjos tecnológicos de regulação adotados durante a pandemia de COVID-19 empregados em contextos diversos, com objetivos relativos à garantia de acesso. Os elementos que compõem cada uma das categorias identificadas estão apresentados no Quadro 2, sendo possível observar que vários artigos aparecem em mais de uma categoria de arranjos tecnológicos de regulação.

### Reorganização de serviços

Os arranjos categorizados como “Reorganização de serviços” envolvem em sua natureza organizacional/operacional essencialmente processos de trabalho e transitam fortemente na esfera micropolítica, com predomínio do uso de tecnologias leves ^32^.

Foram identificados 32 artigos que representam 71% do total dos resultados, sendo assim, devido à abrangência desta categoria, é possível observar variedade da população-alvo, localidade da realização do estudo e tipos de arranjos de reorganização de serviços. Em relação à população-alvo, a maior parte dos estudos (40%) se refere aos serviços destinados ao tratamento de COVID-19, seja para pacientes suspeitos ou com diagnóstico confirmado ^2^ ^,^ ^5^ ^,^ ^33^ ^,^ ^34^ ^,^ ^35^ ^,^ ^36^ ^,^ ^37^ ^,^ ^38^. Na sequência, os estudos relacionados a procedimentos/cirurgia eletiva representam 18% do total, outros tipos de população-alvo também foram listados, como os pacientes de psiquiatria, pediatria, cardiologia, cirurgia vascular, cabeça e pescoço, entre outros.

Quanto aos tipos de arranjos tecnológicos de regulação encontrados na categoria “Reorganização de serviços”, foi possível agrupar as experiências em seis subcategorias: (a) implantação de fluxos/protocolos, diferenciados entre protocolos de triagem, de admissão, de transferência, de segregação de paciente e internos; (b) alteração da oferta, incluindo reduções, ampliações e readequações para a manutenção da oferta; (c) readequação no processo de trabalho, distribuídos entre treinamentos realizados, realocação de profissionais, alteração em procedimentos de assistência e coleta de dados epidemiológicos; (d) readequação estrutural, identificadas como reestruturação de espaços físicos, separação de alas e mudança na destinação de determinados ambientes para atender a demanda; (e) estruturação de central de transferência/regulação, alocados entre implantação de central de transferência, centralização da gestão da central e estabelecimento de sistema de referências específico para atender as necessidades impostas pela pandemia; e a (f) requisição de leitos privados.

Ficou evidente que certos arranjos, como a implementação de fluxos e protocolos, concentram grande parte dos estudos, frequentemente como uma adaptação nos processos de trabalho, em consonância com outros arranjos, como a expansão da oferta. Contudo, é fundamental ressaltar que a maioria dos arranjos enquadrados nessa categoria fez uso de recursos já existentes, com um foco primordial nos processos de trabalho ou na readequação da finalidade de serviços ou alas já existentes.

### Uso de ferramentas virtuais e de inteligência artificial

Os arranjos que compõem a categoria “Uso de ferramentas virtuais e inteligência artificial” se assemelham por terem a tecnologia como divisora de águas no processo de regulação de leitos. Ainda que os artigos contidos nessa categoria também enfatizem a necessidade de reorganização dos serviços e novos arranjos tecnológicos de regulação, o destaque aqui é o uso da tecnologia em si.

Esta categoria é responsável por 44% dos artigos incluídos, totalizando 20, e tem como principal população-alvo pacientes com suspeita ou confirmação de COVID-19 (69%). Foram identificados, ainda, estudos relacionados a portadores de diabetes, gestantes, pacientes de cirurgia ortopédica e em diálise.

Podemos destacar nesta categoria a existência de subcategorias, agrupadas predominantemente pelo tipo de tecnologia utilizada: (a) ferramenta de triagem/regulação para apoio à decisão clínica com uso de inteligência artificial e tecnologias da informação e comunicação (TIC), identificada entre o uso de aplicativos para apoio de decisão de admissão ou alta hospitalar, emprego de algoritmo na previsão de gravidade dos pacientes/tempo de permanência hospitalar e inteligência artificial para triagem e monitoramento de pacientes; (b) uso de telemedicina, tendo sido empregados nas diferentes etapas do cuidado (admissão, monitoramento e alta), incluindo avaliações pré-operatórias e telematriciamento entre hospitais; (c) monitoramento remoto de pacientes, para acompanhamento da evolução do quadro clínico, empregando inteligência artificial no processo de monitoramento/indicação de internação e na implantação de autoexame como parte do processo de monitoramento, e (d) criação de ala virtual, permitindo acompanhar a evolução de determinados pacientes remotamente.

O exame dos artigos destacou o uso de arranjos tecnológicos de regulação mediados por tecnologia em momentos anteriores à pandemia, os quais na ocasião da emergência sanitária sofreram adaptações, incluindo a incorporação de ferramentas tecnológicas em processos implementados no período em razão do cenário.

### Criação de espaços alternativos

E por fim, a categoria “Criação de espaços alternativos” engloba artigos que abordam a criação de estruturas físicas diferenciadas para o atendimento de pacientes durante a pandemia e destaca o papel de elementos denominados “tecnologias duras”, entretanto, a leitura integral dos estudos apresenta dimensões micropolíticas e o emprego de tecnologias leves e leve-duras ^32^ como instrumento de gestão das estruturas físicas criadas, possibilitando a produção da resposta regulatória na crise sanitária.

O mapeamento da literatura mostrou a criação de espaços alternativos utilizados durante a pandemia como alternativa de expansão da capacidade instalada existente, através da construção de hospitais de campanha, utilização de espaços anexos à hospitais e outros serviços, criação de estruturas comunitárias, entre outros arranjos. As estruturas encontradas nesta categoria não somente estiveram a serviço do cuidado com os pacientes infectados pelo vírus da COVID-19, como também dos demais, permitindo a separação da demanda e continuidade do cuidado.

Adicionalmente, é possível identificar o uso de hotéis como estruturas adaptadas para fins “hospitalares”, visando o acolhimento de casos de menor gravidade e segregação de pacientes, tendo a sua importância atrelada à ampliação da oferta e priorização do uso de leitos hospitalares para os casos de maior gravidade. A participação desse tipo de arranjo nas publicações incluídas foi relativamente limitada, abrangendo cerca de 6 artigos. Grande parte dos estudos se concentrou na população-alvo relacionada a COVID-19, composta por indivíduos com suspeita ou confirmação do diagnóstico, os estudos incluídos nesta categoria também contribuíram significativamente em termos de resultados. Outros aspectos também foram identificados com a implantação das estruturas, como redução na necessidade de internação ^39^ ^,^ ^40^, diminuição no tempo de permanência ^41^, manutenção operacional dos atendimentos não relacionados à COVID-19 ^42^ e o uso dessas estruturas como fonte de receita importante para hotéis ^43^.

## Discussão

À medida que exploramos os resultados deste estudo, observa-se a emergência de um cenário complexo e multifacetado, que lança luz sobre a produção dos arranjos tecnológicos de regulação em meio à pandemia e as possibilidades de expansão do acesso e reorganização dos sistemas de saúde. Esse panorama levanta questões cruciais em relação à importância dos arranjos tecnológicos de regulação para o enfrentamento da pandemia, os desafios em relação à sua continuidade e um olhar acerca da equidade, com base na concepção de regulação do acesso que baseia o estudo.

O questionamento explorado por esta revisão, que trata do uso de arranjos tecnológicos de regulação durante a pandemia, evidenciou a importância da implementação destes mecanismos como forma de oferecer resposta à urgência da crise sanitária, bem como sua eficácia na ampliação do acesso aos serviços de saúde em um contexto marcado por incertezas e pelo rápido crescimento de casos. Sob a ótica da gestão da demanda, são elementos consideráveis: a diminuição no volume de internações ou busca pelo serviço de urgência através do redirecionamento da demanda ^6^ ^,^ ^41^ ^,^ ^44^ ^,^ ^45^ ^,^ ^46^ ^,^ ^47^ ^,^ ^48^ e a redução do tempo de permanência ^41^ ^,^ ^49^ ^,^ ^50^ como resultado da implantação de arranjos tecnológicos de regulação, utilizando monitoramento remoto, inteligência artificial, implantação de protocolos e fluxos específicos na gestão eficaz da demanda.

Em relação à oferta, embora tenha sido demonstrado que houve redução de procedimentos eletivos e não urgentes ^14^ ^,^ ^25^ ^,^ ^34^ ^,^ ^35^ ^,^ ^51^ ^,^ ^52^ ^,^ ^53^, bem como aumento do tempo de espera para determinados procedimentos ^51^, muitos dos arranjos tecnológicos de regulação implantados contribuíram com a manutenção de outras ações de saúde não relacionadas à COVID-19 ^42^ ^,^ ^49^ ^,^ ^54^ ^,^ ^55^ ^,^ ^56^ ^,^ ^57^ ^,^ ^58^, com o aumento da resolutividade ^53^, otimização da oferta ^59^ ^,^ ^60^ e de ampliação do número de leitos ^2^ ^,^ ^14^ ^,^ ^34^.

Outro elemento importante foram os hospitais de campanha, que se apresentaram como mais um dispositivo na construção da resposta e podem ser considerados como um dos arranjos tecnológicos de regulação amplamente utilizados na ocasião da pandemia, conforme apontado pela literatura ^61^ ^,^ ^62^ ^,^ ^63^ ^,^ ^64^ ^,^ ^65^. Podem ser definidos como uma instalação de assistência médica temporária de rápida configuração para responder às emergências ou desastres. Além disso, os relatos de experiência apontam para a importância dessas estruturas nos períodos de pico da transmissão, com importantes considerações a respeito das dificuldades enfrentadas no funcionamento, apesar da escassez de recursos materiais e profissionais. Contudo, embora este possa ser considerado um arranjo tecnológico de regulação relevante, não foi identificado através do mecanismo de busca empregado na construção dessa revisão, o que pode reforçar a falta de uniformidade dos descritores apontada anteriormente.

Alguns estudos também relataram que a implantação de centrais de transferências e gestão de leitos que utilizam tecnologia em saúde digital pode ser considerada um arranjo tecnológico de regulação essencial para a melhoria da gestão da oferta e da demanda durante a pandemia ^5^ ^,^ ^33^ ^,^ ^36^ ^,^ ^52^ ^,^ ^66^. Mas é fundamental salientar que a maioria dos arranjos desenvolvidos por meio do uso de tecnologia durante a pandemia tinha algum tipo de estrutura utilizada em momentos anteriores à emergência da pandemia, o que facilitou a adequação e acelerou o processo de readequação em muitos casos.

Em relação aos arranjos tecnológicos de regulação baseados na implantação de novos processos de trabalho e na reorganização dos serviços, a estrutura organizacional existente, bem como os processos e procedimentos implantados foram utilizados como ponto de partida para o desenvolvimento de novos fluxos, protocolos e ações visando a ampliação do acesso ^14^ ^,^ ^25^ ^,^ ^40^ ^,^ ^43^ ^,^ ^51^ ^,^ ^52^ ^,^ ^56^ ^,^ ^59^ ^,^ ^67^. Esta categoria de arranjos tecnológicos de regulação se articula com o conceito de regulação produtora de cuidado, utilizando tecnologias leves e relacionais, promovendo comunicação mais fluida no interior dos serviços e estabelecendo experiencias de cuidado vivo “em ato” ^22^ ^,^ ^23^ ^,^ ^68^.

Algumas medidas, como a designação de equipes específicas, em contexto que exigiu a gestão de crise no estabelecimento de articulação local e regional ^5^ ^,^ ^33^ ^,^ ^66^, e a implantação de centrais de regulação e transferência de pacientes ^5^ ^,^ ^36^ ^,^ ^52^ ^,^ ^59^, incluindo a nomeação de comitês e coordenadores, demonstram uma tendência na centralização dos processos, que aceleram modificações e uniformizam ações, sendo reconhecidas como benéficas para o enfrentamento da crise sanitária.

Os resultados aqui evidenciados apontam para uma série de fatores essenciais que contribuíram significativamente para a adaptação bem-sucedida dos serviços de saúde durante a pandemia. Entre esses, destacam-se a flexibilidade na organização dos serviços, a agilidade na tomada de decisões, o aprimoramento da comunicação entre profissionais de saúde, bem como o surgimento de uma cultura de solidariedade e empatia.

A adaptação dos arranjos tecnológicos de regulação durante a pandemia proporcionou valiosas lições, entretanto, a transformação de estratégias em algo duradouro exigirá um esforço contínuo e um foco na regulamentação, financiamento e equidade, visando a construção de sistemas de saúde mais resilientes e capazes de responder com eficácia a desafios futuros. Os diferentes modelos de governança empregados globalmente também se constituem como importante desafio na consolidação de arranjos de forma mais regionalizada e articulada no bojo dos sistemas de saúde existentes.

Embora não haja uma solução única que se aplique a todas as circunstâncias, a identificação dos elementos que contribuíram para o processo pode ser um legado importante da pandemia, entretanto, para avançar nessa reflexão, é necessário indagar até que ponto podemos continuar inovando ao implementar arranjos tecnológicos de regulação dinâmicos, baseados em uma regulamentação ágil que conecta os serviços de saúde, incluindo profissionais e gestores, às necessidades dos usuários?

Dentre as limitações deste estudo, identificamos a insuficiência de informações, nos estudos primários, dos desdobramentos e da continuidade dos arranjos tecnológicos de regulação implantados. Ainda que alguns autores tenham mencionado a importância dos arranjos implantados para o enfrentamento de novos desafios sanitários, bem como para o cotidiano dos serviços ^53^ ^,^ ^54^ ^,^ ^69^, não é possível mensurar quais destes arranjos permanecem em operação e os efeitos produzidos. Um bom exemplo é a telessaúde que teve crescimento significativo de uso durante a pandemia ^7^, porém, com o seu arrefecimento apresentou diminuição nos níveis de utilização ^44^.

Outra limitação do estudo diz respeito à diversidade de sistemas de saúde compreendidos nesta revisão. Partindo de características fundamentais da composição dos sistemas de saúde como por exemplo o tipo de cobertura, o exercício da governança e o financiamento, alcançando o olhar para o uso e acesso, as diferenças encontradas devem ser consideradas ao analisar de forma mais profunda as respostas produzidas globalmente.

## Conclusão

A pressão sobre os sistemas de saúde ocasionada pela gravidade e altíssima letalidade da COVID-19 evidenciou e potencializou a necessidade e urgência de se estar pronto para a ação. Este cenário favoreceu a implantação de novos arranjos tecnológicos de regulação, que também contaram com algum grau de abrandamento de regras e procedimentos burocráticos, bem como uma maior autonomia administrativa.

Cabe ressaltar que para os sistemas de saúde mais consolidados, a capacidade de resiliência, utilizando recursos desenvolvidos em momentos anteriores à pandemia de COVID-19, foi maior, e possibilitou não apenas o desenvolvimento de uma melhor resposta, mas a capacidade de desenvolver instrumentos e processos já existentes para um novo cenário desafiador.

Entretanto, ainda que os novos arranjos utilizados na condução da crise tenham demonstrado resultados positivos como a ampliação da oferta, a agilidade no acesso, emprego de tecnologia para viabilizar o cuidado, entre outras, a manutenção destes arranjos tecnológicos de regulação no cotidiano dos serviços de saúde inclui a necessidade de superar barreiras de regulamentação, estabelecer de padrões de qualidade, segurança e financiamento sustentável. Para as várias estratégias já existentes que sofreram adaptações para o cenário pandêmico, é preciso endereçar estas questões para que se possa vislumbrar ações de longo prazo, quando aplicáveis.

Além disso, é preciso encarar a falta de uniformidade na definição do próprio conceito de regulação do acesso no panorama global, considerando que a fragmentação dos sistemas de saúde que ocasiona fragilidade na resposta a emergências é um problema real, necessário e de enfrentamento urgente para alçar o objetivo de tornar os sistemas de saúde cada vez mais resilientes. Por outro lado, é oportuno reconhecer a potência da regulação do acesso como ferramenta de produção do cuidado em saúde, levando em consideração a força dos processos micropolíticos no cuidado em saúde e da centralidade do usuário na produção do cuidado, ainda que considere o modelo médico hegemônico existente, constituindo um importante campo de disputa.

A questão da equidade também não pode ser negligenciada. Embora essas inovações tenham mostrado seu valor, é essencial garantir que a melhoria no acesso aos serviços de saúde seja equitativa, principalmente quando consideramos a pluralidade de comunidades e grupos populacionais em seus diferentes territórios, com construções culturais muito particulares. Considerar o âmago micropolítico multifacetado destes processos é fundamental no combate às iniquidades no acesso aos serviços de saúde observadas em sistemas de países marcados fortemente pela desigualdade social, como é o caso daqueles situados no Sul Global.

É fundamental o aprofundamento na identificação e qualificação de arranjos e lições aprendidas durante a COVID-19, que se estabeleceram de forma mais consolidada nos sistemas de saúde no cenário pós pandêmico, visto que esta qualificação auxiliará em análises para outros cenários complexos de saúde vindouros.
